# Supplementary material for: Drug repurposing for aging research using model organisms
Source: Aging Cell. 2017 Jun 16;16(5):1006–15. doi: 10.1111/acel.12626 (PMC5595691; doi:10.1111/acel.12626)
Supplement: Supplementary file 7 — Data S1 Zip‐Archive of all report cards. [file ACEL-16-1006-s007.zip › RC_ACY.pdf]

ACY

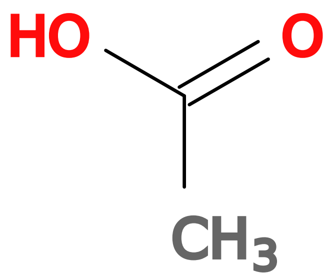

Database identifiers

ChEMBLCompound	CHEMBL539
CHEBI	15366
eMolecules	475727

Ranking

	Rank	Score
Drosophila	471/697	0.303
C. elegans	482/591	0.086

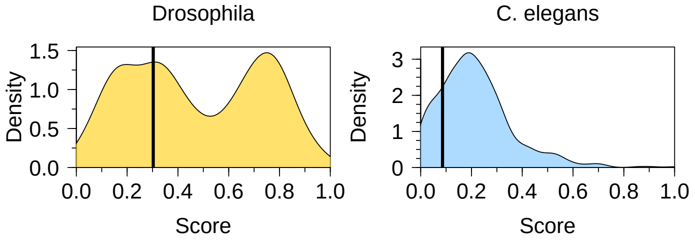

	Ageing implication	Domain conservation	Binding site conservation	Binding affinity	Bioavailability	Lipinski	Promiscuity	Purchasability	Drug approval	**Total**
Drosophila	1.0	0.972	1.0	0.335	(0.9)	0.0	-0.191	0.1	0.1	0.303
C. elegans	1.0	0.967	0.933	0.335	0.253	0.0	-0.191	0.1	0.1	0.086

Names

- **acetic acid**
- Glacial
- Acetasol
- acide acetique
- AcOH
- Ch3co2h
- E-260
- Essigsaeure
- Ethylic acid
- HOAc
- INS No. 260
- MeCOOH

· Methanecarboxylic acid
· Orlex
· Vosol

Roles

food acidity regulator, protic solvent, Daphnia magna metabolite, antimicrobial food preservative

Status

Approved drug (according to ChEMBL)	Yes
Number of Rule of 5 violations	0
Binding affinity to original target in log units (RF-Score prediction)	4.32
Burns *C. elegans* bioavailability prediction	-1.94

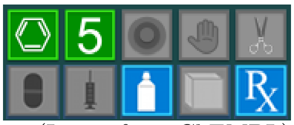

(Image from ChEMBL)

Compound Target Characteristics

Myeloperoxidase

Best gene implication in ageing for this target family came from gene D3ZYH8 annotated in UniProt release 2014_02. Annotation GO 7568 (aging) was Inferred from Expression Pattern

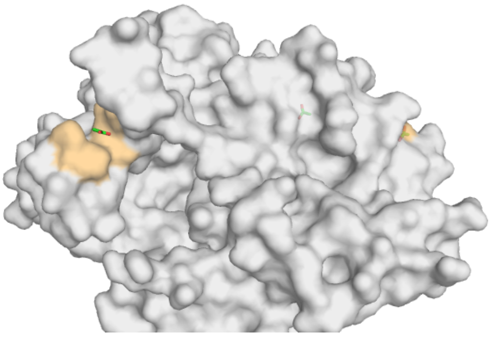

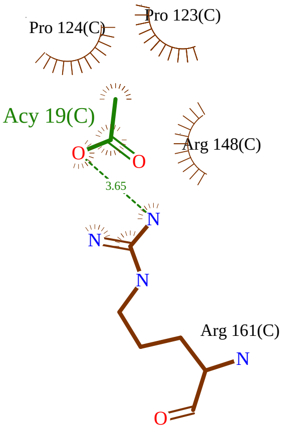

protein	amino acids contacts (binding site)							
PDB:1dnu:chainC:P05164	P	P	R	R	P	R	R	R
PDB:1dnw:chainC:P05164	P	P	R	R	P	R	R	R
sp:P05164:PERM_HUMAN	P	P	R	R	P	R	R	R
tr:D3ZYH8:D3ZYH8_RAT	Q	P	R	R	P	K	P	-
sp:P11247:PERM_MOUSE	P	P	R	R	P	K	P	R
tr:Q8MT50:Q8MT50_DROME	P	Y	R	R	K	E	P	L
tr:Q9VEJ9:Q9VEJ9_DROME	P	Y	R	R	K	E	P	L
sp:Q9VEG6:PERC_DROME	F	A	R	G	P	K	E	L
sp:Q23490:MLT7_CAEEL	S	K	R	R	V	K	I	M
tr:O01892:O01892_CAEEL	T	N	R	R	D	T	A	I
tr:O17241:O17241_CAEEL	P	S	R	R	Q	D	P	L

protein	whole protein ident	simil	domain-based ident	simil	contact-based ident	simil
PDB:1dnu:chainC:P05164	1.0	1.0	1.0	1.0	1.0	1.0
PDB:1dnw:chainC:P05164	1.0	1.0	1.0	1.0	1.0	1.0
sp:P05164:PERM_HUMAN	1.0	1.0	1.0	1.0	1.0	1.0
tr:D3ZYH8:D3ZYH8_RAT	0.61	0.68	0.79	0.87	0.5	0.51
sp:P11247:PERM_MOUSE	0.83	0.93	0.87	0.96	0.75	0.85
tr:Q8MT50:Q8MT50_DROME	0.21	0.48	0.28	0.63	0.38	0.51
tr:Q9VEJ9:Q9VEJ9_DROME	0.26	0.65	0.31	0.71	0.38	0.51
sp:Q9VEG6:PERC_DROME	0.22	0.54	0.29	0.67	0.25	0.54
sp:Q23490:MLT7_CAEEL	0.27	0.65	0.34	0.73	0.25	0.58
tr:O01892:O01892_CAEEL	0.22	0.56	0.28	0.68	0.25	0.53
tr:O17241:O17241_CAEEL	0.15	0.41	0.29	0.69	0.38	0.56

Pxt (FBgn0261987) associated phenotypes
female semi-sterile

(Information from FlyBase)

Pxt (UniProt:Q9VEG6) annotation

Function: Required for ovarian follicle maturation. Involved in the formation of a rigid and insoluble egg chorion by catalyzing chorion protein cross-linking through dityrosine formation and phenol oxidase-catalyzed chorion melanization. (PubMed:18216169).
Cofactor: heme bNote=Binds 1 heme b (iron(II)-protoporphyrin IX) group per heterodimer. ;
Subunit: Heterodimer. (UniProtKB:P05164,).
Subcellular location: Secreted (PubMed:18216169). Note=In the chorion layer of the mature eggs.
Tissue specificity: Expressed at low levels in the germarium and early follicles. Expression becomes progressively stronger during vitellogenesis, and is highly expressed in germ cells and somatic cells. A subset of follicle cells, termed border cells (BC), exhibit a high level of expression. (PubMed:18216169).
Developmental stage: Expressed both maternally and zygotically. (PubMed:12459925).
Disruption phenotype: Females are sterile, and maturing follicles show defects in actin filament formation, nurse cell membrane stability and border cell migration. (PubMed:18216169).

(Information from UniProt)

mlt-7 (WBGene00022743) associated phenotypes
dumpy, lethal, sterile

(Information from WormBase)

mlt-7 (UniProt:Q23490) annotation

Function: Plays an essential role in cuticle biogenesis. Required in combination with bli-3 for correct formation of cross-links in cuticle collagens. (PubMed:19406744).
Tissue specificity: Expressed in the hypodermal cells, specifically the head and seam/body. (PubMed:19406744).
Developmental stage: Expressed in all adult and larval stages. Expression is cyclical and coincides with each of the larval molts, peaking at 12, 18, 24, and 30h post-hatching. (PubMed:19406744).
Disruption phenotype: Gross morphological abnormalities causing larval arrest with associated molt defects and low levels of embryonic lethality. Cuticle function and integrity are also impaired. (PubMed:19406744).

(Information from UniProt)

Heat shock protein HSP 90-alpha

Best gene implication in ageing for this target family came from gene Q18688 annotated in UniProt release 2014_02. Annotation GO 8340 (determination of adult lifespan) was Inferred from Genetic

Interaction

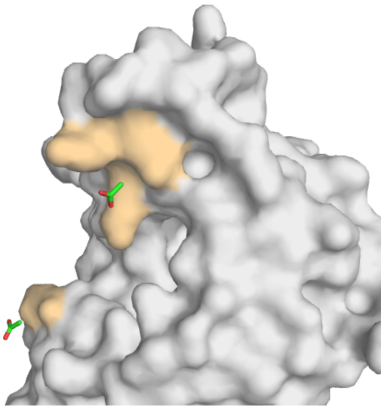

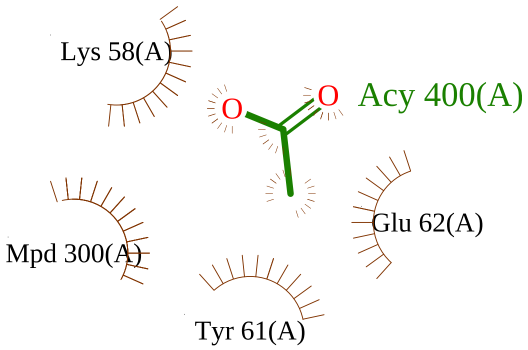

protein	amino acids contacts (binding site)
PDB:1osf:chainA:P07900	K Y E Q
tr:Q86SX1:Q86SX1_HUMAN	K Y E Q
tr:K9JA46:K9JA46_HUMAN	K Y E Q
sp:P07900:HS90A_HUMAN	K Y E Q
tr:Q6B437:Q6B437_RAT	K Y E Q
tr:D3ZXC7:D3ZXC7_RAT	K Y E Q
tr:D3ZSM0:D3ZSM0_RAT	K Y E Q
sp:P82995:HS90A_RAT	K Y E Q
tr:B7ZC50:B7ZC50_MOUSE	K Y E Q
tr:Q8C2A9:Q8C2A9_MOUSE	K Y E Q
tr:Q3TKB9:Q3TKB9_MOUSE	K Y E Q
tr:Q3TKG0:Q3TKG0_MOUSE	K Y E Q
sp:P07901:HS90A_MOUSE	K Y E Q
tr:Q80Y52:Q80Y52_MOUSE	K Y E Q
sp:P02828:HSP83_DROME	K Y E Q
sp:Q18688:HSP90_CAEEL	K Y Q Q
sp:P02829:HSP82_YEAST	K Y K Q

	whole protein		domain-based		contact-based	
protein	ident	simil	ident	simil	ident	simil
PDB:1osf:chainA:P07900	1.0	1.0	1.0	1.0	1.0	1.0
tr:Q86SX1:Q86SX1_HUMAN	0.16	0.16	0.54	0.54	1.0	1.0
tr:K9JA46:K9JA46_HUMAN	1.0	1.0	1.0	1.0	1.0	1.0
sp:P07900:HS90A_HUMAN	1.0	1.0	1.0	1.0	1.0	1.0
tr:Q6B437:Q6B437_RAT	0.22	0.22	0.85	0.85	1.0	1.0
tr:D3ZXC7:D3ZXC7_RAT	0.28	0.32	0.82	0.91	1.0	1.0
tr:D3ZSM0:D3ZSM0_RAT	0.63	0.7	0.89	0.95	1.0	1.0
sp:P82995:HS90A_RAT	0.99	1.0	1.0	1.0	1.0	1.0
tr:B7ZC50:B7ZC50_MOUSE	0.27	0.27	0.86	0.86	1.0	1.0
tr:Q8C2A9:Q8C2A9_MOUSE	0.37	0.37	0.99	1.0	1.0	1.0
tr:Q3TKB9:Q3TKB9_MOUSE	0.75	0.76	0.99	1.0	1.0	1.0
tr:Q3TKG0:Q3TKG0_MOUSE	0.75	0.76	0.99	1.0	1.0	1.0
sp:P07901:HS90A_MOUSE	0.99	1.0	0.99	1.0	1.0	1.0
tr:Q80Y52:Q80Y52_MOUSE	0.99	1.0	0.99	1.0	1.0	1.0
sp:P02828:HSP83_DROME	0.77	0.92	0.85	0.96	1.0	1.0
sp:Q18688:HSP90_CAEEL	0.74	0.89	0.76	0.94	0.75	0.93
sp:P02829:HSP82_YEAST	0.6	0.84	0.68	0.9	0.75	0.87

Hsp83 (FBgn0001233) associated phenotypes

cell cycle defective, cell polarity defective, developmental rate defective, female semi-fertile, lethal - all die before end of pupal stage, lethal - all die during third instar larval stage, partially lethal, partially lethal - majority die, short lived

(Information from FlyBase)

Hsp83 (UniProt:P02828) annotation

Function: Molecular chaperone that promotes the maturation, structural maintenance and proper regulation of specific target proteins involved for instance in cell cycle control and signal transduction. Undergoes a functional cycle that is linked to its ATPase activity. This cycle probably induces conformational changes in the client proteins, thereby causing their activation. Interacts dynamically with various co-chaperones that modulate its substrate recognition, ATPase cycle and chaperone function. Together with Hop and piwi, mediates canalization, also known as developmental robustness, likely via epigenetic silencing of existing genetic variants and suppression of transposon-induced new genetic variation. Required for piRNA biogenesis by facilitating loading of piRNAs into PIWI proteins. (PubMed:21186352, PubMed:22902557).
Subunit: Homodimer (By similarity). Forms a complex with Hop and piwi; probably Hop mediates the interaction between piwi and Hsp83. Interacts with shu. (, PubMed:21186352, PubMed:22902557).
Subcellular location: Cytoplasm.
Induction: In contrast to other major heat shock proteins, this one is also expressed at normal growth temperatures. It is also developmentally expressed during oogenesis.
Domain: The TPR repeat-binding motif mediates interaction with TPR repeat-containing proteins.

(Information from UniProt)

daf-21 (WBGene00000915) associated phenotypes

chemotaxis variant, dauer constitutive, dauer recovery variant, larval lethal, lethal, odorant chemosensory response variant, sluggish, sterile

(Information from WormBase)

daf-21 (UniProt:Q18688) annotation

Function: Molecular chaperone that promotes the maturation, structural maintenance and proper regulation of specific target proteins involved for instance in cell cycle control and signal transduction. Undergoes a functional cycle that is linked to its ATPase activity. This cycle probably induces conformational changes in the client proteins, thereby causing their activation. Interacts dynamically with various co-chaperones that modulate its substrate recognition, ATPase cycle and chaperone function. By stabilizing the receptor-type guanylate cyclase daf-11 or another signal transduction component that regulates cGMP levels, plays a role in dauer formation and chemotaxis to non-volatile and volatile attractants detected by AWC sensory neurons (PubMed:10790386, PubMed:7828815). Participates in the control of cell cycle progression at the prophase/metaphase transition in oocyte development by ensuring the activity of wee-1.3 kinase, which negatively regulates cdk-1 through its phosphorylation (PubMed:16466390). Regulates yap-1 nuclear export after heat shock treatment (PubMed:23396260). (PubMed:10790386, PubMed:16466390, PubMed:23396260, PubMed:7828815).
Subunit: Homodimer. (UniProtKB:P08238).
Subcellular location: Cytoplasm, perinuclear region (PubMed:12950278). Note=Perinuclear region of somatic cells.
Tissue specificity: In the embryo comma stage, expression is strongly detected in cells of the head region and less so in other areas. In early larvae, expressed in postembryonic germ cells derived from Z2 and Z3 cells and the head region, in both hermaphrodites and males. Under heat stress conditions, larval expression is not only detected in germ cells, but also all over the body. In adult hermaphrodites, expression is localized uniquely in the germ cells. (PubMed:12950278).
Developmental stage: Highly expressed throughout development. (PubMed:10790386).
Domain: The TPR repeat-binding motif mediates interaction with TPR repeat-containing proteins.

(Information from UniProt)

Alcohol dehydrogenase 4

Best gene implication in ageing for this target family came from gene A1L128 via mapping the annotation from RGD 71028 annotated in RGD 2014-03-11. Annotation GO 7568 (aging) was Inferred from Expression Pattern

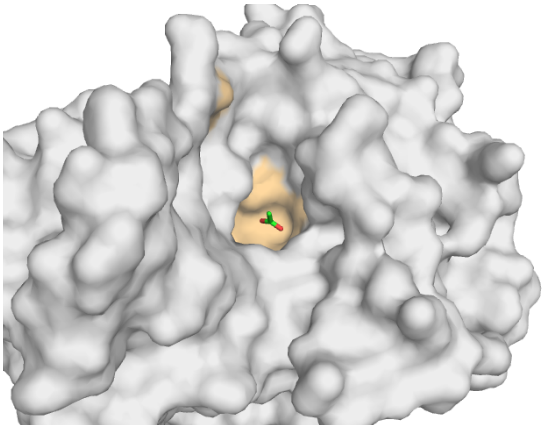

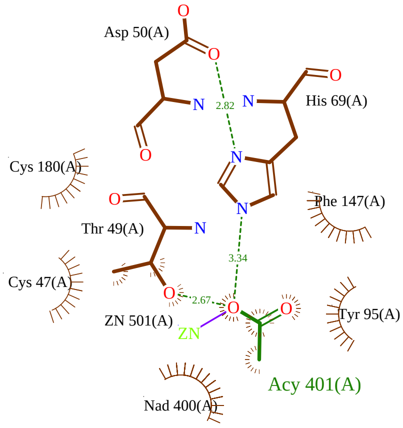

protein	amino acids contacts (binding site)						
PDB:3cos:chainA:P08319	C	T	H	Y	F	C	S
sp:P08319:ADH4_HUMAN	C	T	H	Y	F	C	S
tr:A1L128:A1L128_RAT	C	T	H	F	M	C	E
sp:Q9QYY9:ADH4_MOUSE	C	T	H	F	M	C	K
sp:P46415:ADHX_DROME	C	T	H	Y	M	C	G
sp:Q17335:ADHX_CAEEL	C	T	H	Y	M	C	G
tr:Q965R0:Q965R0_CAEEL	C	T	H	Y	M	C	G
sp:P32771:FADH_YEAST	C	T	H	Y	M	C	G

	whole protein		domain-based		contact-based	
protein	ident	simil	ident	simil	ident	simil
PDB:3cos:chainA:P08319	0.99	1.0	1.0	1.0	1.0	1.0
sp:P08319:ADH4_HUMAN	1.0	1.0	1.0	1.0	1.0	1.0
tr:A1L128:A1L128_RAT	0.72	0.92	0.68	0.9	0.57	0.8
sp:Q9QYY9:ADH4_MOUSE	0.71	0.91	0.69	0.91	0.57	0.73
sp:P46415:ADHX_DROME	0.55	0.85	0.56	0.85	0.71	0.87
sp:Q17335:ADHX_CAEEL	0.51	0.82	0.53	0.83	0.71	0.87
tr:Q965R0:Q965R0_CAEEL	0.33	0.55	0.3	0.51	0.71	0.87
sp:P32771:FADH_YEAST	0.46	0.78	0.48	0.81	0.71	0.87

Fdh (FBgn0011768) associated phenotypes
heat sensitive, memory defective

(Information from FlyBase)

Fdh (UniProt:P46415) annotation
Function: Class-III ADH is remarkably ineffective in oxidizing ethanol, but it readily catalyzes the oxidation of long-chain primary alcohols and the oxidation of S-(hydroxymethyl) glutathione.
Cofactor:Zn(2+)Note=Binds 2 Zn(2+) ions per subunit. ;

(Information from UniProt)

H24K24.3 (UniProt:Q17335) annotation
Function: Class-III ADH is remarkably ineffective in oxidizing ethanol, but it readily catalyzes the oxidation of long-chain primary alcohols and the oxidation of S-(hydroxymethyl) glutathione.
Cofactor: Zn(2+)Note=Binds 2 Zn(2+) ions per subunit. ;
Subunit: Homodimer.

Subcellular location: Cytoplasm

(Information from UniProt)

CELE$_Y 50D4C.2(UniProt : Q965R0)annotation$
Cofactor: Zn(2+) Evidence=(RuleBase:RU361277);

(Information from UniProt)

Insulin-degrading enzyme

Best gene implication in ageing for this target family came from gene P14735 annotated in UniProt release 2014_02. Annotation GO 8340 (determination of adult lifespan) was Inferred from Direct Assay

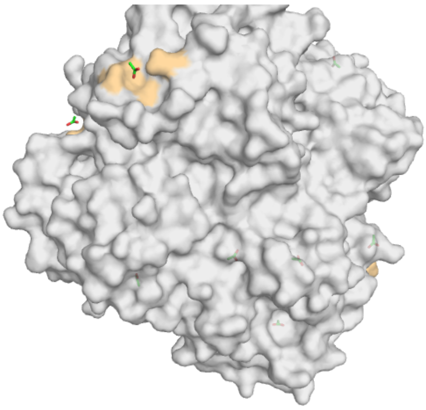

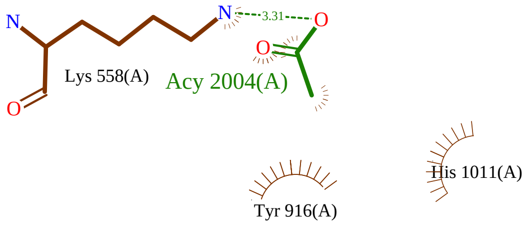

protein	amino acids contacts (binding site)
PDB:3e4a:chainA:P14735	K L E E Y I N T V N V D P E R E F K Y E F G L H
sp:P14735:IDE_HUMAN	K L E E Y I N T V N V D P E R E F K Y E F G L H
sp:P35559:IDE_RAT	K L E E Y I N T V N V D P D R E F K Y E F G L H
sp:P22817:IDE_DROME	K V N R V I S D M V V D P K R S F R Y - F C K F
tr:G5ED87:G5ED87_CAEEL	K V H E I V Q S Q G I E P K K Q Y R Y - L F L A
tr:Q8IA89:Q8IA89_CAEEL	K V D E I V L S Q T M D P K Q E Y R Y - L F L A
tr:Q8MXK1:Q8MXK1_CAEEL	K V D E I V L S Q T M D P K Q E Y R Y - L F L A
tr:O16249:O16249_CAEEL	K V D E I V L S Q T M D P K Q E Y R Y - L F L A
sp:Q06010:STE23_YEAST	K L D E I V K E S D I D P T R E F K Y Q F T L M

	whole protein		domain-based		contact-based	
protein	ident	simil	ident	simil	ident	simil
PDB:3e4a:chainA:P14735	0.99	0.99	0.99	0.99	1.0	1.0
sp:P14735:IDE_HUMAN	1.0	1.0	1.0	1.0	1.0	1.0
sp:P35559:IDE_RAT	0.95	0.99	0.97	0.99	0.96	0.98
sp:P22817:IDE_DROME	0.44	0.79	0.46	0.82	0.38	0.56
tr:G5ED87:G5ED87_CAEEL	0.37	0.75	0.39	0.78	0.21	0.64
tr:Q8IA89:Q8IA89_CAEEL	0.38	0.74	0.4	0.77	0.29	0.6
tr:Q8MXK1:Q8MXK1_CAEEL	0.38	0.76	0.41	0.78	0.29	0.6
tr:O16249:O16249_CAEEL	0.38	0.75	0.4	0.77	0.29	0.6
sp:Q06010:STE23_YEAST	0.36	0.76	0.37	0.77	0.5	0.74

Ide (FBgn0001247) associated phenotypes

decreased cell number, decreased cell size, developmental rate defective, large body, long lived, nutrition conditional, partially lethal - majority die, short lived, size defective, small body

(Information from FlyBase)

Ide (UniProt:P22817) annotation

Function: Can cleave insulin and TGF-alpha. (PubMed:3139025).

Cofactor: Zn(2+)Note=Binds 1 zinc ion per subunit. ;
Biophysicochemical properties: pH dependence: Optimum pH is 7-8. (PubMed:3139025); Temperature dependence: Optimum temperature is 37 degrees Celsius. (PubMed:3139025);
Induction: Inhibited by bacitracin and sulfhydryl-specific reagents. (PubMed:3139025).

(Information from UniProt)

2',3'-cyclic-nucleotide 3'-phosphodiesterase

Best gene implication in ageing for this target family came from gene P13233 annotated in UniProt release 2014_02. Annotation GO 7568 (aging) was Inferred from Expression Pattern

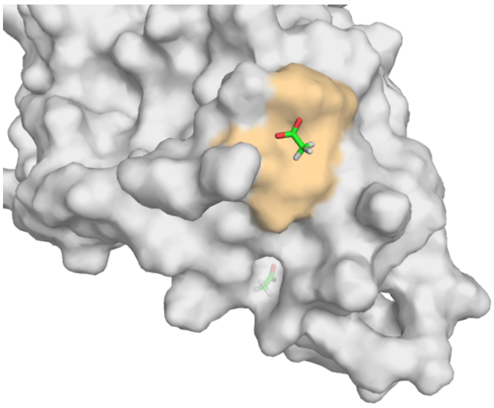

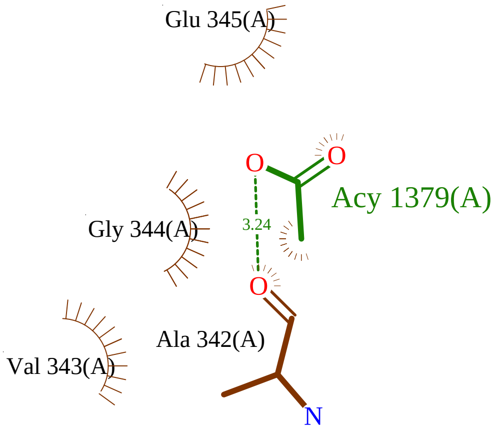

protein	amino acids contacts (binding site)
PDB:2yp0:chainA:P16330	Y G E P K W S L P P G S R A D A V G E
PDB:2ype:chainA:P16330	Y G E P K W S L P P G S R A D A V G E
PDB:3zbz:chainA:P16330	Y G E P K W S L P P G S R A D A V G E
tr:B4DFN6:B4DFN6_HUMAN	Y G E P K W T L P R G S R A D E V G E
sp:P09543:CN37_HUMAN	Y G E P K W T L P R G S R A D E V G E
sp:P13233:CN37_RAT	Y G E P K W S L P P G S R A D E V G E
tr:Q3TYL9:Q3TYL9_MOUSE	Y G E P K W S L P P G S R A D A V G E
sp:P16330:CN37_MOUSE	Y G E P K W S L P P G S R A D A V G E
tr:Q3TYV5:Q3TYV5_MOUSE	Y G E P K W S L P P G S R A D A V G E
tr:Q19804:Q19804_CAEEL	- - - - - - - - - - - - - - - - - - -

	whole protein		domain-based		contact-based	
protein	ident	simil	ident	simil	ident	simil
PDB:2yp0:chainA:P16330	1.0	1.0	1.0	1.0	1.0	1.0
PDB:2ype:chainA:P16330	1.0	1.0	0.99	1.0	1.0	1.0
PDB:3zbz:chainA:P16330	1.0	1.0	0.99	1.0	1.0	1.0
tr:B4DFN6:B4DFN6_HUMAN	0.35	0.42	0.59	0.72	0.84	0.86
sp:P09543:CN37_HUMAN	0.85	0.96	0.8	0.95	0.84	0.86
sp:P13233:CN37_RAT	0.95	0.99	0.95	0.99	0.95	0.94
tr:Q3TYL9:Q3TYL9_MOUSE	0.95	0.95	1.0	1.0	1.0	1.0
sp:P16330:CN37_MOUSE	1.0	1.0	1.0	1.0	1.0	1.0
tr:Q3TYV5:Q3TYV5_MOUSE	1.0	1.0	1.0	1.0	1.0	1.0
tr:Q19804:Q19804_CAEEL	0.11	0.29	0.03	0.05	0.0	0.0

Tyrosine-protein phosphatase non-receptor type 1

Best gene implication in ageing for this target family came from gene P18031 via mapping the annotation from Ensembl ENSG00000196396 via mapping the annotation from EntrezGene 5770 via mapping the annotation from GenAgeHuman 0033 annotated in GenAge release 17.

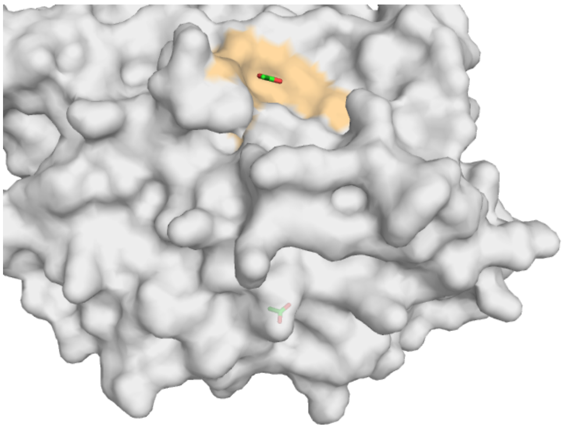

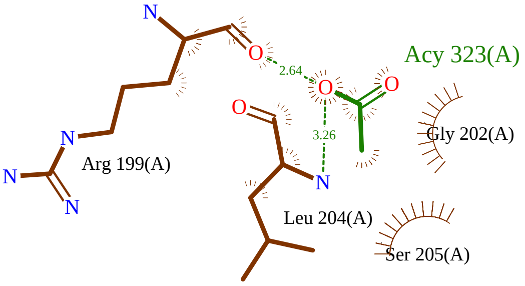

protein	amino acids contacts (binding site)															
PDB:1een:chainA:P18031	Y	R	E	R	R	G	S	L	S	P	L	K	E	R	G	Q
PDB:1pxh:chainA:P18031	Y	R	E	R	R	G	S	L	S	P	L	K	E	R	G	Q
sp:P18031:PTN1_HUMAN	Y	R	E	R	R	G	S	L	S	P	L	K	E	R	G	Q
tr:A8K3M3:A8K3M3_HUMAN	Y	R	E	R	R	G	S	L	S	P	L	K	E	R	G	Q
sp:P20417:PTN1_RAT	Y	R	E	R	R	G	S	L	S	P	L	K	E	R	G	Q
tr:Q3TB93:Q3TB93_MOUSE	Y	R	E	R	R	G	S	L	S	L	L	K	E	R	G	Q
tr:Q3TZW9:Q3TZW9_MOUSE	Y	R	E	R	R	G	S	L	S	L	L	K	E	R	G	Q
sp:P35821:PTN1_MOUSE	Y	R	E	R	R	G	S	L	S	L	L	K	E	R	G	Q
sp:Q9W0G1:PTP61_DROME	Y	E	R	R	R	G	C	L	S	R	L	K	E	R	G	Q
tr:D3DMI1:D3DMI1_DROME	Y	E	R	R	R	G	C	L	S	R	L	K	E	R	G	Q
tr:I0B1P6:I0B1P6_DROME	Y	E	R	R	R	G	C	L	S	R	L	K	E	R	G	Q

	whole protein		domain-based		contact-based	
protein	ident	simil	ident	simil	ident	simil
PDB:1een:chainA:P18031	1.0	1.0	1.0	1.0	1.0	1.0
PDB:1pxh:chainA:P18031	1.0	1.0	1.0	1.0	1.0	1.0
sp:P18031:PTN1_HUMAN	1.0	1.0	1.0	1.0	1.0	1.0
tr:A8K3M3:A8K3M3_HUMAN	1.0	1.0	1.0	1.0	1.0	1.0
sp:P20417:PTN1_RAT	0.84	0.94	0.96	0.99	1.0	1.0
tr:Q3TB93:Q3TB93_MOUSE	0.7	0.73	0.95	0.99	0.94	0.94
tr:Q3TZW9:Q3TZW9_MOUSE	0.83	0.94	0.95	0.99	0.94	0.94
sp:P35821:PTN1_MOUSE	0.83	0.94	0.95	0.99	0.94	0.94
sp:Q9W0G1:PTP61_DROME	0.31	0.59	0.49	0.8	0.75	0.81
tr:D3DMI1:D3DMI1_DROME	0.31	0.59	0.49	0.8	0.75	0.81
tr:I0B1P6:I0B1P6_DROME	0.31	0.59	0.49	0.8	0.75	0.81

Ptp61F (UniProt:Q9W0G1) annotation

Function: Non-receptor protein tyrosine phosphatase required for maintaining Dock in its non-phosphorylated state. (PubMed:12014990, PubMed:8463208).

Subcellular location: Isoform A: Cytoplasm. Endomembrane system. Note=Associates with the membranes of the reticular network and the mitochondria.

Subcellular location: Isoform B: Nucleus.

Tissue specificity: Expressed during oogenesis and embryogenesis. Isoform A and isoform B are expressed in distinct patterns. Isoform B is expressed in the mesoderm and neuroblast layer during germband extension and later in the gut epithelia. Isoform A accumulates in 16 segmentally repeated stripes in the ectoderm during germband extension. These stripes are flanked by, and adjacent to, the domains of engrailed and wingless gene expression in the anterior/posterior axis. In stage 10 embryos, isoform A colocalizes with the area lateral to the denticle belts that will give rise to naked cuticle. Isoform A is also expressed later in embryogenesis in the central nervous system. (PubMed:9256342).

(Information from UniProt)

Dipeptidyl peptidase 1

Best gene implication in ageing for this target family came from gene P80067 annotated in UniProt release 2014_02. Annotation GO 7568 (aging) was Inferred from Expression Pattern

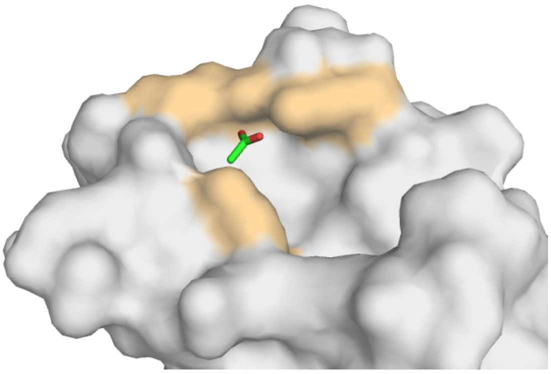

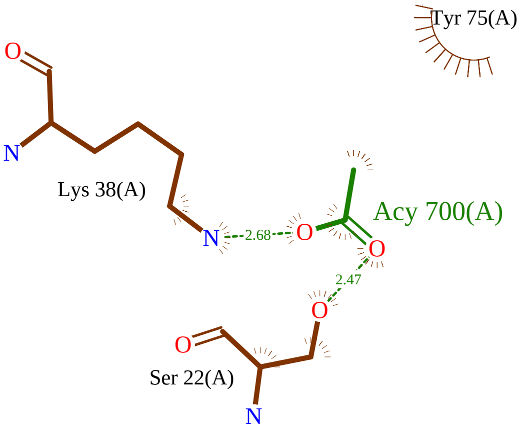

protein	amino acids contacts (binding site)
PDB:2djf:chainA:P53634	S G K Y
sp:P53634:CATC_HUMAN	S G K Y
sp:P80067:CATC_RAT	R H E Y
tr:D3Z220:D3Z220_MOUSE	R S E Y
tr:Q3U9B7:Q3U9B7_MOUSE	R S E Y
sp:P97821:CATC_MOUSE	R S E Y
tr:Q3UBY5:Q3UBY5_MOUSE	R S E Y
tr:P92005:P92005_CAEEL	- - P M

	whole protein		domain-based		contact-based	
protein	ident	simil	ident	simil	ident	simil
PDB:2djf:chainA:P53634	1.0	1.0	1.0	1.0	1.0	1.0
sp:P53634:CATC_HUMAN	1.0	1.0	1.0	1.0	1.0	1.0
sp:P80067:CATC_RAT	0.79	0.93	0.75	0.92	0.25	0.52
tr:D3Z220:D3Z220_MOUSE	0.39	0.49	0.78	0.93	0.25	0.61
tr:Q3U9B7:Q3U9B7_MOUSE	0.76	0.9	0.78	0.93	0.25	0.61
sp:P97821:CATC_MOUSE	0.78	0.93	0.78	0.93	0.25	0.61
tr:Q3UBY5:Q3UBY5_MOUSE	0.78	0.93	0.78	0.93	0.25	0.61
tr:P92005:P92005_CAEEL	0.23	0.55	0.16	0.48	0.0	0.0

Growth factor receptor-bound protein 2

Best gene implication in ageing for this target family came from gene P62994 annotated in UniProt release 2014_02. Annotation GO 7568 (aging) was Inferred from Physical Interaction

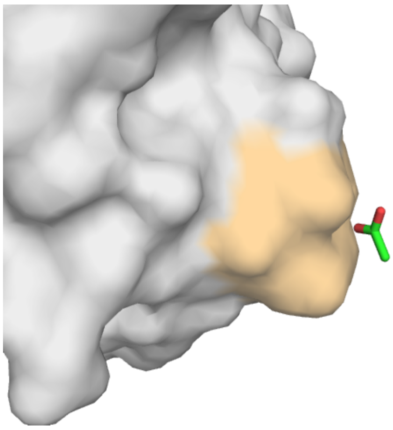

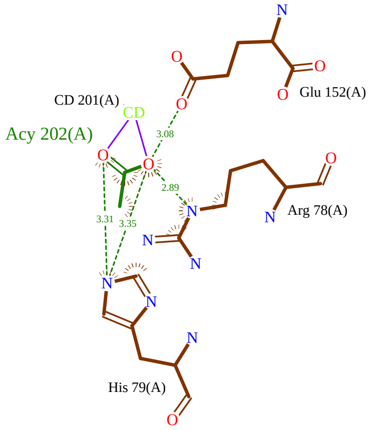

protein	amino acids contacts (binding site)		
PDB:3wa4:chainA:P62993	R	H	E
sp:P62993:GRB2_HUMAN	R	H	E
tr:B0LPF3:B0LPF3_HUMAN	R	H	E
sp:P62994:GRB2_RAT	R	H	E
tr:B1AT92:B1AT92_MOUSE	R	H	E
sp:Q60631:GRB2_MOUSE	R	H	E
tr:Q3U5I5:Q3U5I5_MOUSE	R	H	E
sp:Q08012:DRK_DROME	-	H	I
sp:P29355:SEM5_CAEEL	T	R	N

	whole protein		domain-based		contact-based	
protein	ident	simil	ident	simil	ident	simil
PDB:3wa4:chainA:P62993	0.98	0.99	0.98	0.99	1.0	1.0
sp:P62993:GRB2_HUMAN	1.0	1.0	1.0	1.0	1.0	1.0
tr:B0LPF3:B0LPF3_HUMAN	1.0	1.0	1.0	1.0	1.0	1.0
sp:P62994:GRB2_RAT	1.0	1.0	1.0	1.0	1.0	1.0
tr:B1AT92:B1AT92_MOUSE	0.93	0.94	0.97	0.98	1.0	1.0
sp:Q60631:GRB2_MOUSE	1.0	1.0	0.99	1.0	1.0	1.0
tr:Q3U5I5:Q3U5I5_MOUSE	1.0	1.0	0.99	1.0	1.0	1.0
sp:Q08012:DRK_DROME	0.64	0.86	0.64	0.86	0.33	0.0
sp:P29355:SEM5_CAEEL	0.55	0.81	0.56	0.84	0.0	0.67

drk (FBgn0004638) associated phenotypes

dominant, endocytosis defective, fully, learning defective, lethal - all die before end of pupal stage, memory defective, neuroanatomy defective, rescuable maternal effect, some die during pupal stage

(Information from FlyBase)

drk (UniProt:Q08012) annotation

Function: Required for proper signaling by sevenless. May act to stimulate the ability of Sos to catalyze Ras1 activation by linking sevenless and Sos in a signaling complex. (PubMed:8462097, PubMed:8462098).

Subunit: Interacts with autophosphorylated sev via SH2 domain and Sos, Dos and Dab via SH3 domains. Binds to tyrosine phosphorylated Dab via the SH2 domain. (PubMed:12128212, PubMed:8462097, PubMed:8462098, PubMed:9671493).

Subcellular location: Membrane (PubMed:8462098); Peripheral membrane protein (PubMed:8462098).

Tissue specificity: Found mainly in the developing eye and in the antennal disk. Also observed in other imaginal disks tested and in the embryo.

(Information from UniProt)

sem-5 (WBGene00004774) associated phenotypes

cell migration variant, egg laying variant, hermaphrodite sex muscle development variant, maternal effect lethal emb, rod like larval lethal, sex muscle morphology variant, vulval cell induction variant, vulvaless

(Information from WormBase)

sem-5 (UniProt:P29355) annotation

Function: Acts both in vulval induction and sex myoblast migration. Presumably interacts with the kinase receptor let-23 and with a target that modifies the Ras-like protein let-60.

(Information from UniProt)

Protein kinase C delta type

Best gene implication in ageing for this target family came from gene Q05655 annotated in UniProt release 2014_02. Annotation GO subterm of 7568 (aging) was Inferred from Mutant Phenotype

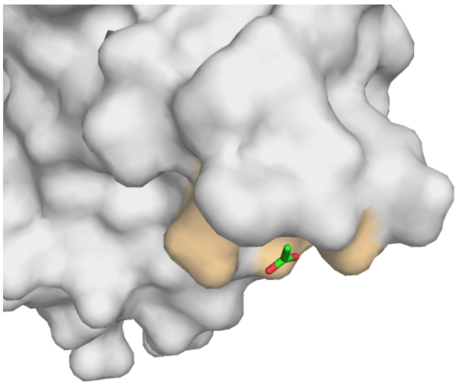

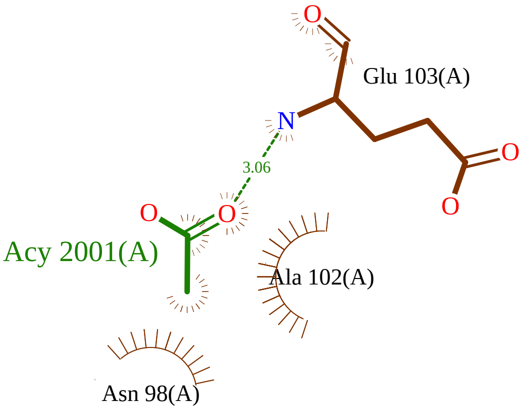

protein	amino acids contacts (binding site)
PDB:1yrk:chainA:Q05655	R N A E
tr:C9J9P1:C9J9P1_HUMAN	R N A E
tr:C9K0E3:C9K0E3_HUMAN	R N A E
tr:B4DFV1:B4DFV1_HUMAN	R N A E
sp:Q05655:KPCD_HUMAN	R N A E
tr:D4A0U0:D4A0U0_RAT	R N A E
tr:Q53YN4:Q53YN4_MOUSE	R N A E
sp:P28867:KPCD_MOUSE	R N A E
tr:Q1EBZ4:Q1EBZ4_DROME	S E E H
tr:Q9VYN1:Q9VYN1_DROME	S E E H
sp:P34722:KPC1_CAEEL	K S T H

	whole protein		domain-based		contact-based	
protein	ident	simil	ident	simil	ident	simil
PDB:1yrk:chainA:Q05655	0.98	0.98	1.0	1.0	1.0	1.0
tr:C9J9P1:C9J9P1_HUMAN	0.18	0.18	1.0	1.0	1.0	1.0
tr:C9K0E3:C9K0E3_HUMAN	0.33	0.33	1.0	1.0	1.0	1.0
tr:B4DFV1:B4DFV1_HUMAN	0.89	0.89	0.48	0.48	1.0	1.0
sp:Q05655:KPCD_HUMAN	1.0	1.0	1.0	1.0	1.0	1.0
tr:D4A0U0:D4A0U0_RAT	0.44	0.5	0.94	0.98	1.0	1.0
tr:Q53YN4:Q53YN4_MOUSE	0.9	0.97	0.96	0.99	1.0	1.0
sp:P28867:KPCD_MOUSE	0.9	0.97	0.96	0.99	1.0	1.0
tr:Q1EBZ4:Q1EBZ4_DROME	0.02	0.07	0.23	0.67	0.0	0.5
tr:Q9VYN1:Q9VYN1_DROME	0.13	0.25	0.23	0.67	0.0	0.5
sp:P34722:KPC1_CAEEL	0.45	0.75	0.33	0.78	0.0	0.76

tpa-1 (WBGene00006599) associated phenotypes

antimicrobial gene expression variant, cell stress response variant, drug induced gene expression variant, isothermal tracking behavior variant, level of protein expression variant, phorbol ester resistant, response to injury variant

(Information from WormBase)

tpa-1 (UniProt:P34722) annotation

Function: Diacylglycerol (DAG)-dependent serine/threonine-protein kinase that phosphorylates a range of cellular proteins (By similarity). Phosphorylates mlk-1, a component of the JNK pathway. Involved in axon regeneration after injury probably by activating the JNK pathway (PubMed:23072806). (, PubMed:23072806).

(Information from UniProt)

Werner syndrome ATP-dependent helicase

Best gene implication in ageing for this target family came from gene Q19546 annotated in UniProt release 2014_02. Annotation GO 8340 (determination of adult lifespan) was Inferred from Mutant Phenotype

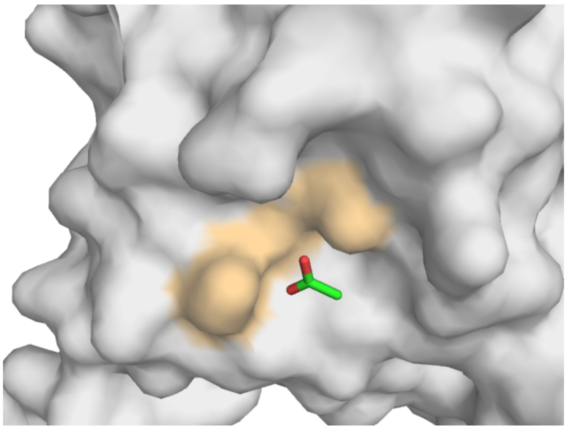

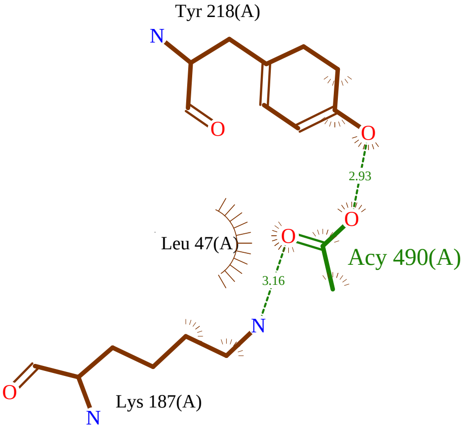

protein	amino acids contacts (binding site)
PDB:2fbt:chainA:Q14191	L K Y
sp:Q14191:WRN_HUMAN	L K Y
tr:M0R8Q0:M0R8Q0_RAT	L K Y
tr:F1LTH9:F1LTH9_RAT	L K Y
tr:Q3TB25:Q3TB25_MOUSE	L K Y
sp:O09053:WRN_MOUSE	L K Y
sp:Q19546:WRN_CAEEL	- - -
sp:P35187:SGS1_YEAST	L S K

	whole protein		domain-based		contact-based	
protein	ident	simil	ident	simil	ident	simil
PDB:2fbt:chainA:Q14191	0.99	1.0	1.0	1.0	1.0	1.0
sp:Q14191:WRN_HUMAN	1.0	1.0	1.0	1.0	1.0	1.0
tr:M0R8Q0:M0R8Q0_RAT	0.35	0.42	0.86	0.97	1.0	1.0
tr:F1LTH9:F1LTH9_RAT	0.69	0.88	0.86	0.97	1.0	1.0
tr:Q3TB25:Q3TB25_MOUSE	0.44	0.55	0.86	0.96	1.0	1.0
sp:O09053:WRN_MOUSE	0.69	0.88	0.86	0.96	1.0	1.0
sp:Q19546:WRN_CAEEL	0.2	0.5	0.12	0.27	0.0	0.0
sp:P35187:SGS1_YEAST	0.18	0.53	0.09	0.38	0.33	0.44

wrn-1 (WBGene00006944) associated phenotypes

organism hypersensitive ionizing radiation

(Information from WormBase)

wrn-1 (UniProt:Q19546) annotation

Function: Essential for the formation of DNA replication focal centers; stably associates with foci elements generating binding sites for RP-A. Exhibits a magnesium-dependent ATP-dependent DNA- helicase activity. May be involved in the control of genomic stability (By similarity).

Subcellular location: Nucleus

(Information from UniProt)
